# Stable Patterns of Gene Expression Regulating Carbohydrate Metabolism Determined by Geographic Ancestry

**DOI:** 10.1371/journal.pone.0008183

**Published:** 2009-12-09

**Authors:** Jonathan C. Schisler, Peter C. Charles, Joel S. Parker, Eleanor G. Hilliard, Sabeen Mapara, Dane Meredith, Robert E. Lineberger, Samuel S. Wu, Brian D. Alder, George A. Stouffer, Cam Patterson

**Affiliations:** 1 McAllister Heart Institute, University of North Carolina, Chapel Hill, North Carolina, United States of America; 2 Division of Cardiology, University of North Carolina, Chapel Hill, North Carolina, United States of America; 3 Expression Analysis, Durham, North Carolina, United States of America; 4 School of Medicine, Duke University, Durham, North Carolina, United States of America; University of Texas Arlington, United States of America

## Abstract

**Background:**

Individuals of African descent in the United States suffer disproportionately from diseases with a metabolic etiology (obesity, metabolic syndrome, and diabetes), and from the pathological consequences of these disorders (hypertension and cardiovascular disease).

**Methodology/Principal Findings:**

Using a combination of genetic/genomic and bioinformatics approaches, we identified a large number of genes that were both differentially expressed between American subjects self-identified to be of either African or European ancestry and that also contained single nucleotide polymorphisms that distinguish distantly related ancestral populations. Several of these genes control the metabolism of simple carbohydrates and are direct targets for the SREBP1, a metabolic transcription factor also differentially expressed between our study populations.

**Conclusions/Significance:**

These data support the concept of stable patterns of gene transcription unique to a geographic ancestral lineage. Differences in expression of several carbohydrate metabolism genes suggest both genetic and transcriptional mechanisms contribute to these patterns and may play a role in exacerbating the disproportionate levels of obesity, diabetes, and cardiovascular disease observed in Americans with African ancestry.

## Introduction

Cardiovascular diseases (CVD) are multifactorial conditions with strong genetic and environmental influences [1], [2]. Despite many advances in diagnosis and treatment, significant challenges remain in understanding, treating and possibly preventing these conditions [3]. Most forms of CVD are multi-factorial, influenced by genetic predispositions as well as environmental factors. On a genetic level, the contribution of any single gene is often small, making investigations of candidate genes difficult to draw any conclusions towards the etiology of CVD [4], [5]. Initial attempts to characterize the underlying causes of CVD have identified a plethora of heterogeneous risk factors including: demographic factors such as family history of premature CVD, gender, and race; behavioral factors including smoking, diet, and activity level; metabolic/biochemical factors related to adiposity, plasma homocysteine, cholesterol levels; and the presence of co-morbid conditions (for example diabetes and hypertension). Whereas individual risk factors often lack significance in terms of predictive power for any given illness, assessment of several risk factors allows appropriate medical interventions both for prevention and treatment of CVD [6].

The study of ancestry and genetics is a highly controversial subject [7], [8], [9]. However, studies have shown that Americans of African ancestry have up to a 2.5-fold increased risk of developing type 2 diabetes, five-fold increased risk of CVD, and eight-fold increase in mortality from CVD compared to Americans of European ancestry [10], [11]. The molecular basis for the increased frequency of these disease occurrences in Americans of African ancestry remains unclear and cannot be adequately explained by social marginalization or various theories of access to health care [1], [11], [12].

The purpose of this study was to identify differential transcriptional signals associated with CVD susceptibility and ancestry. Using genetic samples obtained from a cohort of subjects undergoing cardiac-related evaluation, a strict algorithm that filtered for genomic features at multiple levels identified 151 differentially-expressed genes between Americans of African ancestry and those of European ancestry. Many of the genes identified were associated with glucose and simple sugar metabolism, suggestive of a model whereby selective adaptation to the nutritional environment differs between populations of humans separated geographically over time. These observations represent promising preliminary data indicating that gene expression profiles can be used to phenotypically describe ancestral populations. Furthermore, the data offer at least one potential explanation for the rising incidence of obesity, type 2 diabetes, metabolic syndrome and CVD in the American population as a whole.

## Materials and Methods

### Study Guidelines and Processing

Subjects were enrolled in the University of North Carolina Institutional Review Board approved “SAMARA” study (IRB 04-MED-471). Exclusion criteria included pregnancy, lymphoma, leukemia, chronic immunosuppressive therapy, infection with HIV or HCV, history of solid organ transplant, and anemia. Blood was drawn early in the day from fasted subjects to minimize signals associated with nutritional and diurnal cycle and processed within fifteen minutes. Plasma samples were obtained and RNA and DNA recovered from leukocytes using a modified one-step acid guanidinium thiocyanate-phenol-chloroform extraction (RNA-STAT60, Tel-Test, TX).

### Microarray and qRT-PCR Analysis

Labeled cRNA was co-hybridized to Agilent G4112A Whole Human Genome 44K oligonucleotide arrays with equimolar amounts of Cyanine-3 labeled Universal Human Reference RNA (UHRR, Stratagene, LaJolla, CA) as previously described [13]. Complete, MIAME-compliant datasets were deposited with the Gene Expression Omnibus of the National Center for Biotechnology Information and can be accessed through GEO Series accession number GSE12959. Ten micrograms of total RNA was reverse transcribed into cDNA using the High Capacity cDNA Reverse Transcription Kit (ABI, Applied Biosystems, Framingham, MA) and quantitative real-time PCR (qRT-PCR) reactions were performed using the ABI PRISM® 7900 HT sequence detection system, software and reagents; see Table S1 for primer and probe information. RNA input was calibrated with *18S* expression levels and relative mRNA levels were normalized to levels from the UHRR.

### Genotype Analysis

DNA labeling, hybridization, and data extraction were performed by the DNA Array Core Facility at The Scripps Research Institute (Jupiter, FL). The Genome-Wide Human SNP Array 6.0 (Affymetrix®) was used for hybridizations. Identification of local elements associated with expression (eQTLs) was performed with linear modeling tools in the software package R. For a given gene, all SNPs within 10 kb of the untranslated region were tested. Each SNP was tested by grouping the expression values based on the genotype and assuming an additive relationship between number of ‘B’ alleles and expression level. The genes were selected for differential expression between ancestries, and PCA illustrated segregation of ethnicities based on the genotypes. This combination may inflate the theoretical number of false positives from the linear model. In order to minimize bias, the eQTL procedure was repeated after randomizing the gene-SNP pairs. After 100 such randomizations these permuted statistics were compared to actual statistics in order to estimate the empirical false discovery rate at each theoretical p value threshold. This permutation procedure is specific for identifying local-acting SNPs since it assumes no distant-acting SNPs, and thus is a conservative estimate in the presence of the potential selection bias.

### Statistical Methods

Microarray data were normalized via the loess local intensity normalization method of Smyth and Speed [14], and probes were filtered for features having a normalized intensity of <30 aFU in both channels. Probes were removed if <70% of the data were present across all samples. Missing data points were imputed using the k nearest-neighbors algorithm (k = 17). 18,375 probes passed these filters, and were subsequently used for analysis. Scripts written in the R Statistical Language and Environment (“R”; Version 2.2.1, build r36812, release date 2005-12-20.) and Perl (ActiveState Perl 5.8.1, build 807, release date 2003-11-6) were used to standardize (μ = 0, σ = 1) the data set. Samples were tested for processing time-dependent correlation with gene expression and found to be clear of any technical confounding variables [15]. Furthermore, to avoid any potential analysis bias, ancestry was not associated with subject ID number. Lists of differentially expressed genes were identified using the statistical analysis of microarray algorithm [16] (SAM, Version 2.21, release date 2005-8-24; typical false discovery rate of 1% and 10%), and custom R scripts written in our laboratory. Unsupervised, semi-supervised, and supervised clustering analysis were performed on gene lists essentially as described [17] using Cluster (Version 2.11, http://rana.lbl.gov/EisenSoftware.htm). Heatmaps of cluster analyses were visualized with JavaTreeView (Version 1.0.12, release date 2005-3-14; http://sourceforge.net/projects/jtreeview/) [18]. Nearest centroid classification was performed by calculating two centroids, or vectors of the class mean (AA or CAU) of each gene. Test cases were assigned the class of the most similar centroid as measured by Euclidean distance.

### Plasma Fructosamine Assays

Plasma fructosamine levels were determined using the Kamiya Biosciences (Seattle, WA) Fructosamine Assay Kit, following the manufacturer's recommended protocol. Ten microliters of archived plasma from each subject were utilized for analysis.

### Immunoblotting

Plasma protein concentration was determined for each archived plasma sample (Bio-Rad Quick Start Bradford Assay, Bio-Rad, Hercules, CA). Twenty-five micrograms of total protein were reduced, denatured, and resolved on 4–12% NuPAGE® Novex Bis-Tris Gels (Invitrogen, Carlsbad, CA) in the MES/SDS buffer system. Proteins were transferred to PVDF membranes, reacted with chicken anti-human haptoglobin (NB300-330, Novus, Littleton, CO) and detected with rabbit anti-chicken IGY HRP-conjugate (Sigma, St. Louis, MO). Bands were visualized with Pierce ECL Substrate (Pierce, Rockford, IL). Relative levels of haptoglobin were quantified using Image J (NIH, Bethesda, MD).

## Results

### Demographics and Covariates Analyses

One hundred and sixty-three subjects referred to cardiology services at UNC between the ages of 18 and 50 years enrolled in Phase One of the SAMARA (Supporting a Multi-disciplinary Approach to Researching Atherosclerosis) study were used for this analysis. Using unsupervised clustering and principal components analysis, the variation in gene expression data among the study subjects resulted in a binary segregation of subjects based on self-reported race, either “African American” (AA) or “Caucasian” (CAU). Exclusion of gender and coronary artery disease as confounding factors limited the initial analysis to a “discovery set” of 17 AA and 30 CAU subjects, with equal contributions of gender per cohort.

Within the discovery set of subjects, four demographic variables differed significantly in AA versus CAU subjects: lower smoking pack years and hematocrit levels, and higher occurrence of hypertension and fructosamine levels (Table 1). These findings are in line with other studies performed in the United States that report increased diagnosis of hypertension and decreased mean hematocrit values and smoking rates in Americans of African ancestry versus those of European decent [1], [11], [19].

**Table 1:** Demographic variables in the discovery set of subjects.

Variable	p-value	AA (n = 17)	CAU (n = 30)
Hypertension†	0.037	82.40%	46.70%
Fructosamine (mM/L)‡	0.033	1.90±0.04	1.68±0.07
Hematocrit (%)‡	0.032	37.59±1.20	40.79±0.66
Pack Years‡	0.034	8.26±2.90	18.75±3.83

Categorical† and continuous‡ variables are expressed as percentage of population group or mean±standard error and differences were considered significant at p<0.05, calculated by Fisher's Exact Test, or Student's T Test, respectively.

To test if these demographic variables confounded the analysis of gene expression within the discovery set, we investigated gene expression patterns associated with hematocrit levels, smoking pack-years, hypertension, or fructosamine. A two-class SAM analysis compared the bottom quartile subjects to top quartile subjects and negatives to positives for the continuous and categorical variables, respectively. This method failed to identify any differentially expressed genes (false discovery rate <20%). Alternatively, performing SAM as a quantitative analysis on the continuous variables yielded the same results, indicating these clinical and demographic features are unlikely to impair detection of distinct ancestral transcriptional profiles.

### Differences in Glucose Homeostasis

Despite the numerous studies associating increased rates of metabolic syndrome in persons of African descent, there was no significant difference in clinical diagnosis of diabetes mellitus or mean fasting plasma glucose between AA and CAU subjects (data not shown). We used the measurement of plasma fructosamine as a surrogate marker for functional diabetes, using a threshold value of 2.6 mM/L [20]. Fructosamine measures the concentration of glycated protein adducts in the blood to assess regulation of glucose levels in the diabetic patient over a time period of weeks. Consistent with clinical diagnosis and fasting blood glucose data there was no significant difference between AA and CAU subjects in the number of subjects with fructosamine levels above threshold. However, when fructosamine was analyzed as a continuous variable, we identified significantly higher concentrations in AA compared to CAU subjects (Table 1), suggesting a sub-clinical predisposition to dysglycemia in AA subjects. Overall, the observed differences in fructosamine and other variables (Table 1) within the discovery set of this study agrees with previously published reports on the same topic, implying that, although the number of cohorts in each group was relatively small, the two study groups used in this report are largely representative of their respective populations in the United States. Importantly, the lack of correlation between fructosamine levels and gene expression across our subjects lessens the probability of long-term glucose homeostasis impairment confounding ancestry-dependent expression analyses.

### Identification of Transcriptional Expression Patterns Associated with Ancestry

**Figure 1:**
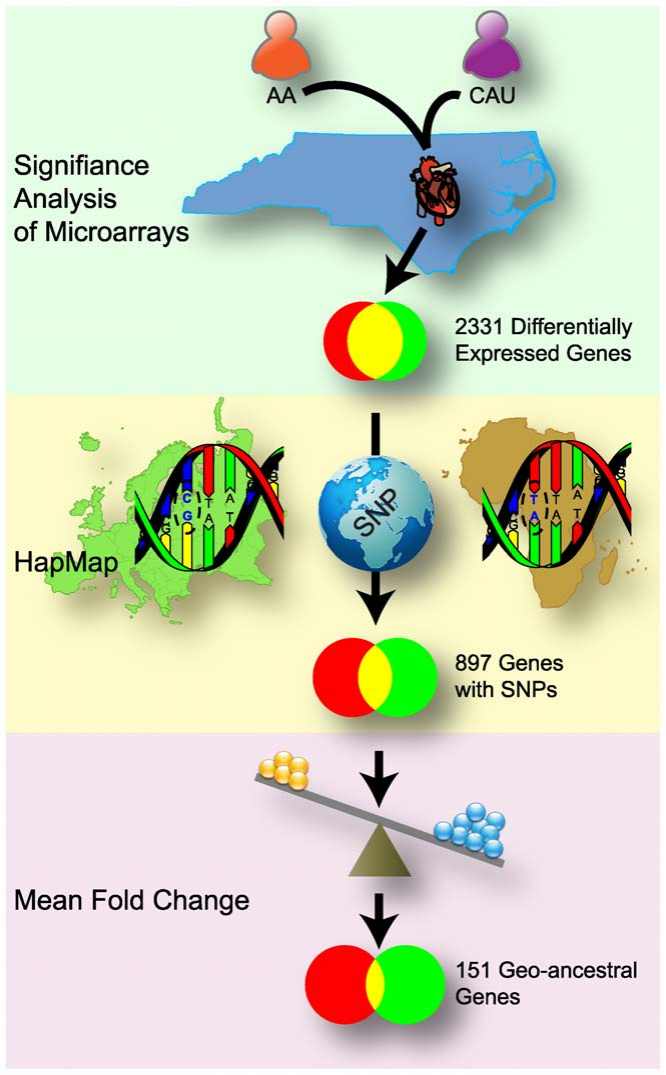
Workflow diagram to identify geo-ancestral genes. The analysis used to identify geo-ancestral genes involved three primary steps: 1) Significance of Microarray (SAM) analysis of two distinct populations in North Carolina, Americans of African or European ancestry, identified 2531 genes as differentially expressed between the populations (**green**); 2) The set of 2531 genes was further restricted to those genes than had SNPs that distinguished to representative ancestral populations from the HapMap project, a total of 897 genes (**yellow**); 3) Further restriction to only those genes that have an absolute mean fold change of 1.3 yielded the set of 151 geo-ancestral genes (**purple**). SNP graphic courtesy of David Hall.

In this discovery set, the SAM statistical technique [16] identified 2521 probes, corresponding to 2331 genes, that were significantly differentially expressed between CAU and AA groups, using a false discovery rate of 1% (Figure 1, Table S2). Given this large number of differentially expressed genes between the study groups, we refined these data by concentrating our focus on genetic differences that had been identified previously between similar populations represented in the HapMap project. The HapMap project is a collection of genetic differences, *i.e.* single nucleotide polymorphisms (SNP), that have been identified between human populations of different geographical regions [21]. Using this approach, we identified the differentially expressed genes from the SAM analysis that contained at least one SNP (within 10kb of the untranslated regions) that distinguishes two HapMap populations with similar ancestral origins as our AA and CAU study groups, the Yoruba people in Ibadan, Nigeria (abbreviation: YRI) and the CEPH population (Utah residents with ancestry in northern and western Europe, abbreviation: CEU), respectively. This analysis uncovered 897 genes (of the 2331 differentially expressed genes in the discovery set, Figure 1) that had single nucleotide polymorphisms (12,276 total SNPs) that were statistically different between YRI versus CEPH populations (p value<1.25E-07, Bonferroni's corrected p value of 0.01, Table S2). Further refining the 897 gene list to those genes that had an absolute mean fold change (MFC) cutoff of greater than 1.3 in our discovery set resulted in the identification of 151 genes; we define these genes as “*geo-ancestral genes*” as they encompass both geographical and ancestral-based transcriptional characteristics (Figure 1, Tables 2 and 3).

**Table 2:** Genes expressed lower in Americans of African versus European ancestry.

Δ	Gene Symbol	GBID	Δ	Gene Symbol	GBID	Δ	Gene Symbol	GBID
−2.79	*S100P*	NM_005980	−1.43	*SNX27*	NM_030918	−1.35	*TTRAP*	NM_016614
−2.49	*SAMD10*	NM_080621	−1.43	*HOXB2*	NM_002145	−1.34	*FLOT1*	NM_005803
−2.08	*PGM1*	NM_002633	−1.43	*PPP2R5A*	NM_006243	−1.34	*ABCA7*	NM_033308
−1.98	*MMP9*	NM_004994	−1.42	*GPR97*	NM_170776	−1.34	*HTATIP2*	NM_006410
−1.96	*HP*	NM_005143	−1.42	*STX10*	NM_003765	−1.34	*GPR160*	NM_014373
−1.96	*EXOSC6*	NM_058219	−1.42	*TP53I11*	BC071606	−1.34	*DHRS8*	NM_016245
−1.87	*C20orf3*	NM_020531	−1.42	*PKD1-like*	NM_024874	−1.34	*FBXL5*	NM_033535
−1.85	*ORM1*	NM_000607	−1.41	*FLJ13052*	NM_023018	−1.34	*DKFZp762O076*	NM_018710
−1.85	*UHSKerB*	NM_021046	−1.41	*HIST1H2AI*	NM_003509	−1.33	*TXN*	NM_003329
−1.81	*CKLFSF1*	NM_181294	−1.41	*IGF2R*	NM_000876	−1.33	*RAF1*	NM_002880
−1.76	*COL9A3*	NM_001853	−1.41	*MME*	NM_007289	−1.33	*REPS2*	NM_004726
−1.69	*BMX*	NM_001721	−1.40	*SNX11*	NM_013323	−1.33	*C20orf24*	NM_018840
−1.68	*QPCT*	NM_012413	−1.39	*HEBP2*	NM_014320	−1.33	*LBR*	NM_194442
−1.67	*DIRC1*	NM_052952	−1.39	*NS3TP1*	NM_019048	−1.33	*MOSPD2*	NM_152581
−1.65	*GPT*	NM_005309	−1.39	*CHI3L1*	NM_001276	−1.33	*SLC40A1*	NM_014585
−1.64	*RAI16*	NM_022749	−1.39	*IFNGR2*	NM_005534	−1.33	*ANPEP*	NM_001150
−1.55	*ASGR2*	NM_001181	−1.39	*LOC120224*	NM_138788	−1.33	*PYGL*	NM_002863
−1.54	*LCE2A*	NM_178428	−1.38	*GCA*	NM_012198	−1.33	*GAB2*	NM_080491
−1.52	*ANXA3*	NM_005139	−1.38	*HIST3H2A*	NM_033445	−1.33	*DREV1*	NM_016025
−1.51	*KRT23*	NM_173213	−1.37	*ATP6V1B2*	NM_001693	−1.33	*DEGS*	NM_003676
−1.50	*USP10*	NM_005153	−1.37	*SEPX1*	NM_016332	−1.32	*SIAT7B*	NM_006456
−1.50	*NOV*	NM_002514	−1.37	*SIAT10*	NM_006100	−1.32	*ChGn*	NM_018371
−1.50	*PPT1*	NM_000310	−1.37	*COPS2*	NM_004236	−1.32	*TPD52L2*	NM_199360
−1.49	*PPP1R12B*	NM_002481	−1.37	*OGFRL1*	NM_024576	−1.32	*PLAU*	NM_002658
−1.49	*HK2*	NM_000189	−1.36	*ASAH1*	NM_004315	−1.31	*CDA*	NM_001785
−1.49	*PGD*	NM_002631	−1.36	*PLAUR*	NM_001005377	−1.31		AC093582
−1.48	*SULF2*	NM_198596	−1.36	*WIPI49*	NM_017983	−1.31	*PAIP2*	NM_016480
−1.47	*MYBPH*	NM_004997	−1.36	*F5*	NM_000130	−1.31	*MGC11324*	NM_032717
−1.47	*C7orf19*	NM_032831	−1.36	*ACOX1*	NM_007292	−1.30		NM_001024688
−1.46	*LAMP2*	NM_013995	−1.35	*STX3A*	NM_004177	−1.30	*MAP4K4*	NM_145687
−1.46	*LMOD1*	NM_012134	−1.35	*RNF135*	NM_197939	−1.30	*CHPT1*	NM_020244
−1.44	*LRWD1*	NM_152892	−1.35	*HIST2H2*	NM_003516	−1.30	*PCTP*	NM_021213
−1.44	*CCPG1*	NM_020739	−1.35	*SRPK1*	NM_003137	−1.30	*GALNAC4S-6ST*	NM_015892
−1.44	*HIST1H2AD*	NM_021065	−1.35	*UBN1*	NM_016936			
−1.44	*IFRD1*	NM_001550	−1.35	*GADD45A*	NM_001924			

Data expressed as Log_2_ mean fold change (Δ). GenBank identifications (GBID) are provided.

**Table 3:** Genes expressed higher in Americans of African versus European ancestry.

Δ	Gene Symbol	GBID	Δ	Gene Symbol	GBID	Δ	Gene Symbol	GBID
1.30	*CRIP1*	NM_001311	1.34	*CCL4*	NM_002984	1.46	*IGJ*	NM_144646
1.30	*FGFR1OP*	NM_194429	1.34	*MTR*	NM_000254	1.47	*TNFRSF17*	NM_001192
1.31		NM_016171	1.34		I_3554426	1.50	*CD19*	NM_001770
1.31		I_3544621	1.35	*RPL30*	NM_000989	1.50	*RPL8*	NM_033301
1.32	*TM4SF9*	NM_005723	1.36	*MYLK*	NM_053025	1.51	*SMAD1*	NM_005900
1.32	*KI0746*	NM_015187	1.36	*LOC127253*	NM_138467	1.52	*C21orf81*	NM_153750
1.32	*RORA*	NM_134260	1.36	*NKG7*	NM_005601	1.60	*CPNE5*	NM_020939
1.32	*ZCCHC7*	NM_032226	1.36		NM_002304	1.67		AY372690
1.33	*FLJ32001*	NM_152609	1.36		AL080251	1.67	*TCL1A*	NM_021966
1.33		NG_001019	1.38		NM_001620	1.70	*GNG11*	NM_004126
1.33	*MMD*	NM_012329	1.38	*GZMH*	NM_033423	1.71	*POU2AF1*	NM_006235
1.34	*POMC*	NM_000939	1.39	*CCL3*	NM_002983	1.77		XM_371884
1.34	*TAF3*	XM_291729	1.41		XM_209178	1.81	*IGHG2*	BC040042
1.34	*LIMS1*	NM_004987	1.41	*RPS15*	NM_001018	1.85		NR_002225
1.34	*RPL24*	NM_000986	1.41	*GNAZ*	NM_002073	1.91		I_3584237
1.34	*SLC12A7*	NM_006598	1.44	*ZNF234*	NM_006630	4.08	*PSPHL*	AJ001612

Data expressed as Log_2_ mean fold change (Δ). GenBank identifications (GBID) are provided.

This approach of filtering the large amount of genetic data originally pulled from our discovery set yielded results that align with findings from other groups. Park *et al.* used a nearest shrunken centroids methodology to identify SNPs that were unique to each of the populations studied in the HapMap project, identifying thousands of ethnically variant SNPs [22]. When we compared our data to the results of this study we found that approximately half of the 897 differentially-expressed ancestral genes, and 71 of the 151 most strongly differentially expressed genes contained “ethnically variant SNPs” identified by Park, *et al.*; suggesting that the delineation of AA and CAU subjects in this study was accurate (see Table S2). Other studies identified genetically linked gene expression differences between various HapMap populations [23], [24]. However, comparing the compilation of Stranger *et al.* and Spielman *et al.* to our findings results in only a 9% overlap (see Table S2); therefore, the integrative approach of filtering gene expression data from AA and CAU subjects from North Carolina with existing SNP databases representing African and European populations both confirm findings from previous studies as well as identify new patterns of gene expression not previously associated with ancestry.

### Similarities in Allele Frequencies between Discovery Set and Respective HapMap Populations

**Figure 2:**
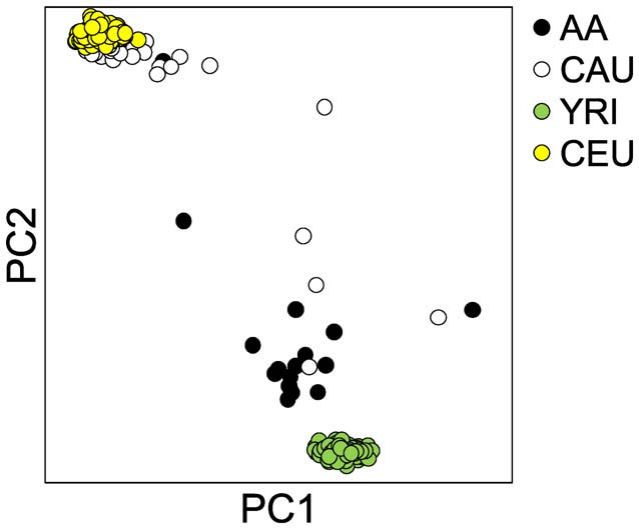
Genomic similarities between North Carolinian and HapMap populations. Unsupervised principal component analysis on genotyping data from the AA and CAU discovery set subjects (n = 17 and 34, respectively) and samples from each corresponding HapMap population, YRI and CEU (n = 90). Principle component 1 and 2 accounted for 22.7% and 11.6%, respectively, of the variation between all four populations.

Previous studies demonstrate the utility and transferability of genetic data from the four HapMap populations to distant ancestral-related populations around the world [25], [26], [27]. Likewise, we used the assumption that the ancestry of AA and CAU subjects in this study was similar to the YRI and CEPH populations, respectively, to generate our list of geo-ancestral genes. However, to test that this assumption was correct, DNA from our discovery set was genotyped using the Affymetrix® Genome-Wide Human SNP Array 6.0, which allowed comparison of principle component analysis of our data with 90 representative samples from each of the YRI and CEU populations. Sorting by the first and second component identified 26 of 30 CAU subjects as more similar to the CEPH versus YRI population and AA subjects (Figure 2). Likewise, 16 of 17 AA subjects associated more with YRI population than the CEPH population and CAU subjects. The alignment of our CAU and AA study cohorts with CEPH and YRI populations previously identified by the HapMap study once again lends credence to accuracy of ethnic identification in the present study. Furthermore, it validates the extensive genetic information in the HapMap database while providing a suitable resource as an ancestral filter for the data set used in this study.

### Quantitative Verification of the Differential Expression of Geo-Ancestral Genes

**Figure 3:**
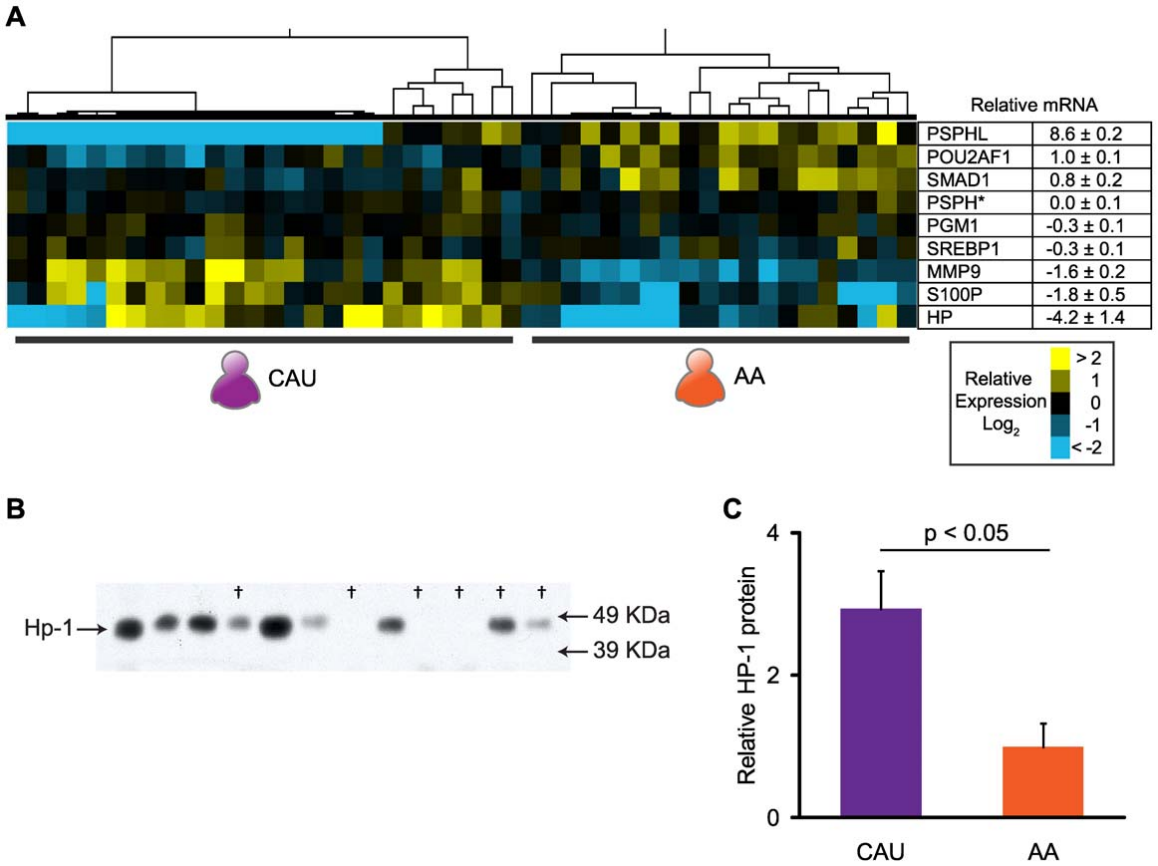
Confirmation of differential gene expression. To verify actual changes in gene expression identified in our analysis, a selected number of genes were measured by Quantitative real-time PCR (qRT-PCR) and/or immunublot analysis. **A**) Results of qRT-PCR analysis of the discovery set subjects normalized to the Universal Human RNA Reference (*left*, heatmap) or as the mean fold change between AA and CAU discovery set cohorts (*right*, table) n = 17 and 34, respectively. All data represented in Log_2_. The differences between AA and CAU subjects were considered significant at p<0.05 for all mRNAs shown, except for *PSPH* (indicated by *). **B**) Immunoblot analysis of Haptoglobin (Hp) in plasma protein samples from randomly selected AA and CAU discovery set subjects (AA samples indicated by †). Immunoreactive bands were observed at the predicted molecular weight, 46 kDa. **C**) Densitometry analysis presented as the relative amount of Haptoglobin±SEM (n = 6 per group) results in a 2.9±0.5 fold increase in Haptoglobin protein in plasma from CAU versus AA subjects.

Quantitative real-time polymerase chain reaction (qRT-PCR) and immunoblot analysis on discovery set samples was used to verify that the geo-ancestral genes identified in our analysis of the microarray data reflect true changes in gene expression. In general, the direction of change in mRNA levels agreed completely with the microarray analysis, but with larger mean fold differences (Figure 3A and Table S2). One exception was the expression of *PSPH*. Microarray analysis indicated that *PSPH* and a similar gene, *PSPHL*, were expressed higher in AA compared to CAU subjects. However, the Agilent array probe for *PSPH* (A_23_P251984) cannot distinguish between these two transcripts. Using qRT-PCR probes specific for each transcript thereby allowed us to determine that *PSPHL* (but not *PSPH*) mRNA levels were differentially expressed between the two groups. Moreover, qRT-PCR could not detect *PSPHL* transcript in most CAU subjects, whereas most AA subjects expressed levels of *PSPHL* transcript near the levels of expression seen in the Universal Human Reference RNA (Figure 3A), indicating near-Boolean expression patterns of the *PSPHL* gene between AA and CAU subjects.

To determine if changes in mRNA can be used to identify potential quantifiable markers in blood samples from the study subjects, we measured circulating levels of the plasma protein, haptoglobin (*HP*). Haptoglobin is an abundant acute-phase reactant elevated in a variety of inflammatory conditions and functions by modulating oxidative damage as well as the salvage of free hemoglobin *via* uptake through the macrophage CD163 scavenger receptor [28], [29]. Western blot analysis of total plasma isolated from the subjects used in our study revealed a 2.9±0.5 fold increase in circulating HP in CAU versus AA subjects (Figure 3B), consistent with both microarray and qRT-PCR analysis (Table S2, Figure 3A). Ancestral-based differences in the levels of plasma haptoglobin are well described in the literature, and correlate with a multitude of genetic distinctions: allelic differences in the coding regions of *HP* [28], SNPs in the upstream promoter sequences [30], and intronic regulatory elements [31]. Importantly, a number of recent studies implicate the absolute amount and quality of the *HP* gene product as an independent risk factor for a multitude of diseases including: diabetes [32]; atherosclerosis [33]; poor clinical outcome following myocardial infarction [28], [34]; and percutaneous coronary interventions [34], [35]. In all of these cases, lower levels of functional haptoglobin increase the likelihood of developing diabetes and cardiovascular disease.

### Validation of Ancestral Patterns of Gene Expression

**Figure 4:**
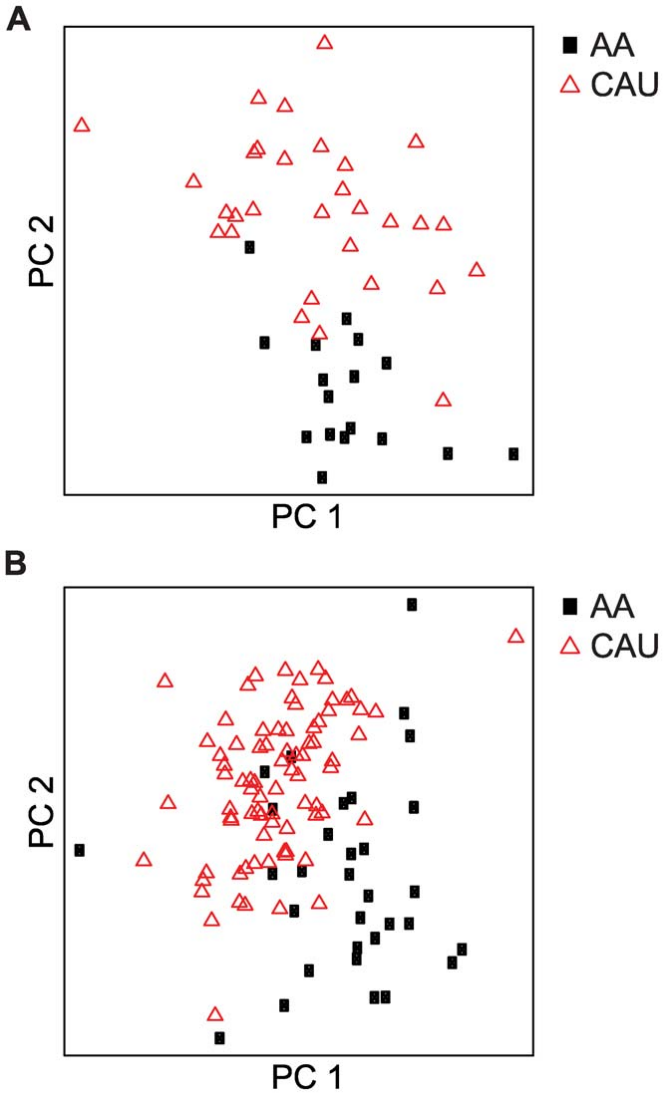
Validation of geo-ancestral genes. The 151 geo-ancestral genes were used to perform supervised principle components analysis of the discovery set of 47 subjects (**A**) and the validation set of 112 unrelated subjects (**B**). The first and second principle components effectively segregated the AA and CAU populations in both cases.

In order to determine how predictive our geo-ancestral gene set was of the general population, we used an independent validation set comprised of 112 unrelated subjects, similarly classified by self-reported ancestry (32 AA and 80 CAU), to validate the 151 geo-ancestral genes. A two-tailed Student's T test identified 102 of the 151 genes (67.5%) as differentially expressed at a p value of ≤0.05 (range: p = 8.32×10^−16^ (*PSPHL*) to p = 4.96×10^−2^ (*STX3A*); Table S2). Furthermore, using the 151 genes for supervised principle component analysis, AA and CAU subjects successfully separated both discovery and validation sets. As expected, principal component analysis successfully grouped the discovery set subjects, with less than 7.0% misclassification (1/17 AA and 2/30 CAU, Figure 4A). Parallel analysis on the validation set led to a similar level of ancestral discrimination in the independent subjects (Figure 4B). A simple nearest centroid classifier built from all 151 genes yielded 84% accuracy in the validation set. These data validate the gene expression patterns observed in the discovery set of 47 subjects, and demonstrate that these geo-ancestral genes are in fact stable phenotypes in Americans of African and European ancestry. Understanding the functional relationships within this gene set could potentially help in explaining the disproportionate predisposition of CVD and other diseases between these populations, a topic that we explore below.

### Ancestral Differences in Expression of Carbohydrate Metabolic Genes

**Figure 5:**
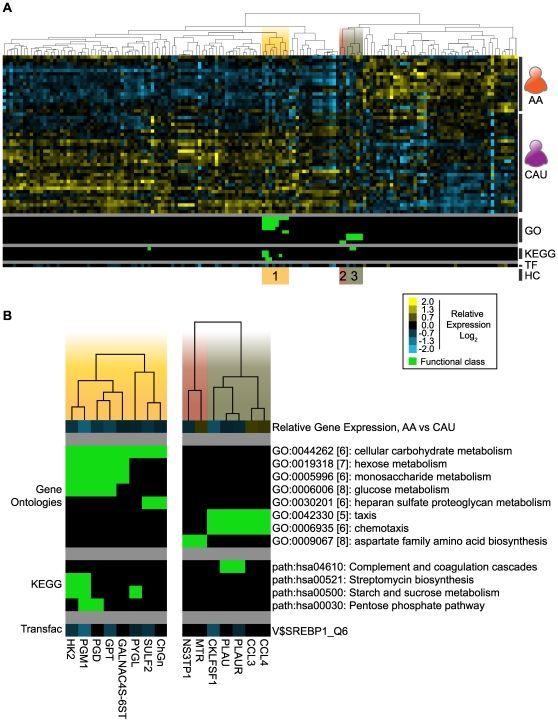
Hyperclustering geo-ancestral genes identify three functional groups. Using the 151 geo-ancestral genes, GATHER identified significantly enriched categories of Gene Ontologies, KEGG pathways and TRANSFAC predicted binding sites. **A**) Hyperclustering of geo-ancestral genes: relative gene expression values are represented by the yellow-blue scale (Log_2_ mean fold change); Inclusion in a functional class of either Gene Ontologies (GO) or KEGG pathways is initiated by green; and predicted TRANSFAC binding sites (TF) are represented as the mean fold change between AA and CAU (using the yellow-blue scale). This resulted in three functional hyperclusters (HC): 1) “*Carbohydrate Metabolism*”; 2) “*Amino Acid Biosynthesis*”; and 3) “*Chemotaxis*”. **B**) Detail showing the average relative gene expression (AA vs CAU) and functional categories for each hypercluster.

Numerous genes expressed at lower levels in AA relative to CAU participate in glucose metabolism (Table 2): primary carbohydrate metabolism (*HK2*, *PYGL*, *GPT*, and *PGM1*); pentose phosphate shunt (*PGD*); and glycosylation of proteins and lipids (*ST3GAL6*, *SULF2*, *GALNAC4S-6ST*, and *ChGn*). Interestingly, the decreased expression of these genes in the AA cohort was notable because of the increased plasma fructosamine levels in these same subjects (Table 1). These results suggest that differences in glucose metabolism between Americans of African and European may reside at the transcriptional level. The *down-regulation* of these genes in the AA cohorts argues against these changes being a compensatory response to hyperglycemia and suggests instead a genetic adaptation to changes in the availability of dietary sugars that may no longer be appropriate to a Western Diet. In order to explore this idea further and to determine the functional importance of the genetic differences we identified, we used hyperclustering analysis of our geo-ancestral gene set to test for differential expression of gene sets that underlie common biological process. Hyperclustering is a method of associating genes with significant enrichments in Gene Ontologies, KEGG pathways, and TRANSFAC analysis [13]. Using this methodology on the 151 geo-ancestral genes, we were able to identify three functional hyperclusters: Carbohydrate Metabolism, Amino Acid Biosynthesis, and Chemotaxis (Figure 5). Of the eight GO categories and four KEGG pathways enriched at a threshold of p≤0.01, half belonged to the Carbohydrate Metabolism hypercluster. These overrepresented KEGG pathways and Gene Ontologies within the Carbohydrate Metabolism hypercluster reaffirm the initial observation of differential expression of carbohydrate metabolic genes, and begin to shed light on factors that may affect glycemic regulation in different ancestral populations.

### Regulation of Geo-Ancestral Genes by the Transcription Factor SREBP1

We next extended our analysis to include algorithms for identifying transcription factor binding sites in the promoter region of differentially expressed genes. This analysis led to the identification of significantly enriched binding sites (p≤0.02) of four predicted transcription factors in the gene set: AML6, HNF3α, E2F1, and SREBP1. Although transcription factor activity can be influenced by several factors, such as post-transcriptional and post-translational modifications and the availability of co-activators and co-repressors, the direction of change in overall activity predicts a complementary change in expression of target genes. The only significant enrichment in either up- or down-regulated target genes of the four transcription factors was SREBP1, exhibiting a 2.9-fold enrichment in down-regulated genes (p<0.05, Table S3). Consistent with this observation, microarray and qRT-PCR analysis identified expression for the gene encoding for SREBP1, *SREBF1*, as significantly decreased by 0.3±0.1-fold in AA relative to CAU subjects (t-test p<0.001, SAM q-value of zero, qRT-PCR p<0.05, Figure 3A, Table S2).

Although SREBP1 was initially characterized as a primary regulator of cholesterol anabolic genes [36], recent studies in animal models detail the critical role SREBP1 plays in the long-term control of both lipid and *glucose* homeostasis in an insulin-dependent manner. As such, SREBP1 mediates the regulation of insulin and glucose responsive genes in a variety of tissues, including skeletal muscle, liver, adipose, and the pancreatic islets of Langerhans [37], [38], [39]. Promoters of five of the eight genes in the carbohydrate metabolic hypercluster (Figure 5) contain SREBP1_Q6 binding motifs. Importantly, while a sequence algorithm identified potential SREBP1 binding sites in these genes, ChIP analysis and DNase footprinting determined SREBP1 *directly interacts* with the promoters and mediates the transcription of both *HKII* [40] and *PGD* [41], which encode the first enzymes in glycolysis and the pentose phosphate pathway, respectively. These data provide a mechanism by which a decrease in SREBP1 expression and transcriptional activity promotes the differential expression of several geo-ancestral genes including multiple carbohydrate metabolic genes.

### The Influence of *cis*-Acting Elements Associated with Gene Expression

Gene expression is influenced by a variety of factors, such as the thousands of common *cis*-acting variations that occur in the population as well as *trans*-acting factors, such as the activity of transcription factors, RNA processing, and signaling molecules [42]. Expression quantitative trait locus (eQTL) analysis combines gene expression and genotyping (*i.e.* SNP) data to determine if changes in gene expression correlate to variations in genomic sequence. We used local eQTL analysis to identify *cis*-acting genetic contributions to the differential expression pattern of the geo-ancestral genes.

**Figure 6:**
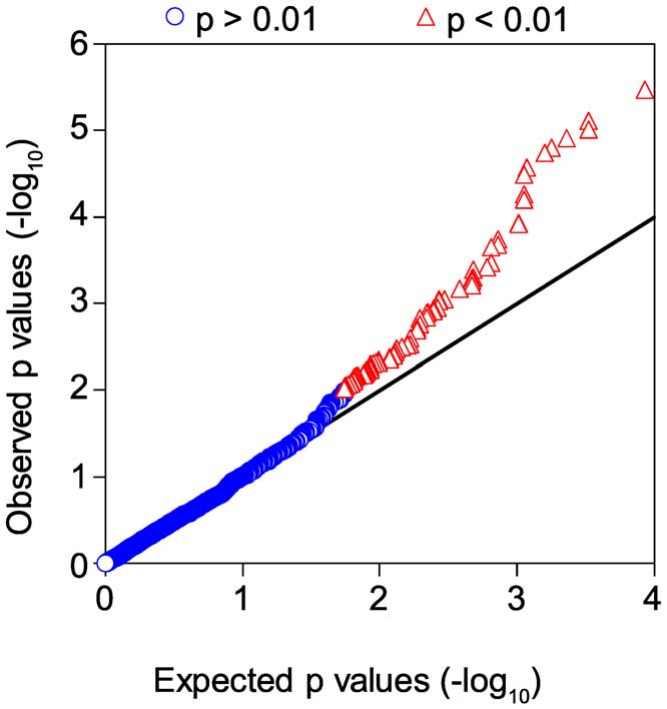
Increase in associations between SNPs and expression of the geo-ancestral genes. The p value of the observed versus predicted eQTLs are plotted using the additive model of association. Data points above the line x = y (--) indicate p values that are smaller than expected due to chance after correcting for selection bias. There were 3241 SNPs found in the 151 geo-ancestral genes, 106 of which associated with expression at a p<0.01 (**red**) with the remainder at p≥0.01 (**blue**).

Differentially expressed genes and SNP associations were both identified with respect to ancestry; as such, the association between genotype and gene expression may be artificially increased (Figure S1). This potential bias was minimized by permutation of the SNP – gene pairs. Association of a SNP with expression after this permutation is assumed to be due to the selection bias. This procedure generates a distribution from which to calculate the expected false discovery rate for a threshold and corresponding set of candidate eQTLs. Comparing the number of observed p values versus expected p values from permutation resulted in more eQTL associations than expected at reasonable thresholds (*e.g.* 16 observed eQTLs compared to 3 expected SNP; FDR = 15.8%, p<0.00025, Table S4). Overall, 119 of the 151 genes were represented by a total of 3241 SNPs, with 106 and 312 SNPs associating with expression or race, respectively (additive or Cochran-Armitage model, p<0.01, Figure 6, and Table S2).

**Figure 7:**
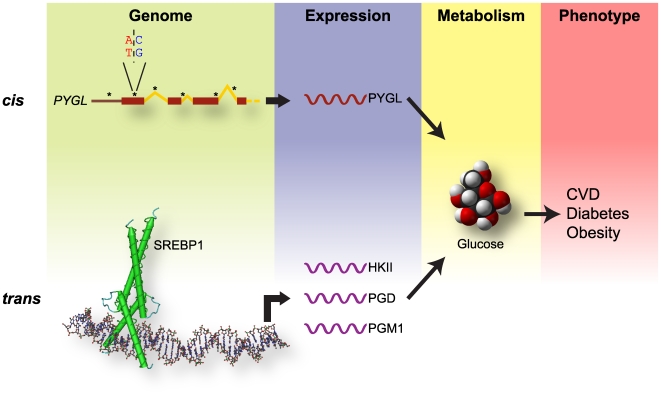
Contributions of *cis*- and *trans*-acting variations to disease pathogenesis. The level of gene expression is influenced by both *cis*- and *trans*-acting factors. Analysis of the carbohydrate metabolic hypercluster identified in the geo-ancestral genes identified both SNPs (*cis*, **top**) and transcription factors such as SREBP1 (*trans*, **bottom**) that function on a genomic level (**green**) contributing to the expression of genes (**blue**) such as *PYGL* and *HKII*. The enzymes encoded by these genes contribute in carbohydrate and glucose metabolism (**yellow**) and likely contribute to the increase the predisposition to multi-factorial diseases (**red**) in Americans of African versus European ancestry.

Local eQTL analysis also allowed us to determine the potential influence of *cis*-acting elements on the differential expression of the previously discussed cadre of carbohydrate metabolic genes. From the eight metabolic genes represented in the Carbohydrate Metabolism hypercluster, four had local eQTL (*CHGN*, *PGM1*, *HK2*, and *PYGL*), and all but *PGD* contained SNPs that associated with race. However, out of this metabolic cluster only *PYGL* had a proportion of eQTL (number of eQTL per total number of gene SNPs, 3.8%, additive model p<0.01) greater than the mean proportion of eQTL from the entire geo-ancestral gene list (3.3%). A similar trend was seen using the proportion of ancestry-associated SNPs (Cochran-Armitage model, Table S2) suggesting that relative to the geo-ancestral list, other factors not defined by these eQTLs may contribute to the differential expression of metabolic genes. In combination with the presence of SREBP1 binding sites in these carbohydrate metabolic genes and the observed decreased in *SREBF1* expression in AA versus CAU subjects, these data suggest that both *trans*-acting elements, such as SREBP1 activity, and hereditary *cis*-acting elements contribute to the differential expression of the carbohydrate metabolic genes identified in this study (Figure 7).

## Discussion

Characterizing inherited patterns of gene transcription is crucial in understanding the meaning of signals related to disease states that vary in incidence across different ancestral populations. This knowledge not only informs the disease data analysis process, it provides important insight into the range of baseline transcriptional regulation in human populations. The International HapMap Project characterizes the scope of genetic differences by genomic sequencing human populations from different geographical areas: Europe, Asia, and Africa. It is important to emphasize that the HapMap Project is highly informative, despite small numbers of subjects from different ancestries: for example, the YRI and CEU datasets derive from 90 total subjects each (30 trios of two parents and an adult child). This effort tabulated millions of single nucleotide polymorphisms within these populations [21]. Several groups have used these data to explore the genetic components of multi-factorial diseases [43], [44]. Recently, whole genome scans identified single nucleotide polymorphisms (SNPs) within the p21.3 region of chromosome 9 that are associated with increased risk of cardiovascular disease and myocardial infarction in Caucasian populations [45], [46], [47]. Although there is no mechanistic data on the association of these non-coding SNPs with disease, it is likely that these silent polymorphisms are associated with transcriptional control of gene expression [48]. The burgeoning correlations between whole-genome SNP patterns and transcriptional regulation is redefining the use of integrative genomics to understand multi-factorial diseases, such as cardiovascular and metabolic diseases [49].

We acknowledge that multi-center genome-wide association studies on cardiovascular disease and diabetes include very large cohorts; however, our approach was designed to better understand disease biology by identifying heritable traits that influence gene expression, not to identify genetic markers solely based on their predictive power of a disease state. Using this approach, the largest transcriptional difference observed in this study was associated with the self-reported ancestry of the subjects. It can be argued that the concept of race, especially self-reported race can be unreliable. However, the correlation between genetic data obtained from our study cohorts respective of self-proclaimed race and data reported from other groups studying similar ancestral populations supports the validity of our cohort partitioning. Indeed, an integrative data analysis, incorporating SNPs identified in the HapMap project, identified differentially expressed genes between Americans of African (AA) and European (CAU) ancestry in the United States that were also structurally distinct between European and African populations (as identified in the HapMap project) that we classified as “*geo-ancestral genes*”. Many of the geo-ancestral genes expressed at lower levels in AA compared to CAU subjects were associated with carbohydrate and glucose metabolism. This subset of genes contained local eQTLs (*cis*-acting) as well as predicted and/or confirmed binding sites for the metabolic transcription factor, SREBP1 (*trans*-acting), also expressed lower in AA subjects (Figure 7). These results are consistent with the observations that Americans of African ancestry are disproportionately affected by obesity, metabolic syndrome, type 2 diabetes, and cardiovascular disease [1] as well as recent studies classifying *SREBF1* as a candidate gene both at an expression and genetic level for these same diseases [50], [51], [52], [53], [54]. Studies suggest that variations at *cis*-regulatory polymorphisms account for more of the population differences in prevalence of complex diseases versus *trans* effects [23], [24], [42]. Likewise, future studies including analysis of *SREBF1* polymorphisms within our study populations and distant eQTL studies to identify other loci that contribute to the regulation of carbohydrate metabolic gene expression should be considered.

A study of the nutritional patterns and diabetes risk among American children demonstrated that, despite better overall compliance with the FDA recommended “Food Pyramid,” American children of African ancestry remained at higher risk for the development of diabetes and pre-diabetic conditions [55]. One interpretation of our findings is that differences in metabolic expression profiles between AA and CAU subjects may not be the sole result of differing nutritional and dietary practices between the study groups. Likewise, diabetics studied within the Seventh Day Adventist Church revealed less benefit for American patients of African versus European ancestry when both groups adhered to the religious dietary practices of the denomination [56]. More focused studies are needed to determine and identify the contribution of genetics to dietary responses, in particular subjects at high risk for multi-factorial diseases such as cardiovascular disease and diabetes. Our study identifies ancestral-dependent patterns of gene expression that may contribute to the differential adaptations of dietary changes and if better understood, could help therapeutically.

## Supporting Information

Figure S1 Illustrating the p-value distributions from different association tests. An eQTL analysis was performed using an additive (left) or genotype (middle) model. In both cases, there is enrichment of small p-values beyond what is expected due to chance. This enrichment is likely due to selection bias because both SNPs and genes were selected based on their association with self reported race.
(0.87 MB TIF)

Table S1 Real-time qPCR reagents. Quadruplicate reactions from each subject's RNA sample were performed (N = 47 subjects; 17 self-identified African American, 30 self-identified Caucasian). RNA input was calibrated with 18S expression levels and relative mRNA levels were normalized to levels from the UHRR (Stratagene, LaJolla, CA). *Determined using ProbeFinder (version 2.44) and the Universal ProbeLibrary (Roche Applied Science, Indianapolis, IN).
(1.39 MB XLS)

Table S2 SNP, gene expression, qRT-PCR, and eQTL analysis.
(1.48 MB XLS)

Table S3 TRANSFAC enrichment analysis. For each predicted TRANSFAC binding site the actual and predicted number (shown in parentheses) are provided assuming an equal distribution between up- and down-regulated genes. * indicates distributions considered unequal at p<0.05, d = fold-enrichment in down-regulated genes.
(1.39 MB XLS)

Table S4 eQTL false discovery rates (FDR) in geo-ancestral genes.
(1.39 MB XLS)
