# Supplementary figures and images for: Stable Patterns of Gene Expression Regulating Carbohydrate Metabolism Determined by Geographic Ancestry
Source: PLoS One. 2009 Dec 9;4(12):e8183. doi: 10.1371/journal.pone.0008183 (PMC2790609; doi:10.1371/journal.pone.0008183)

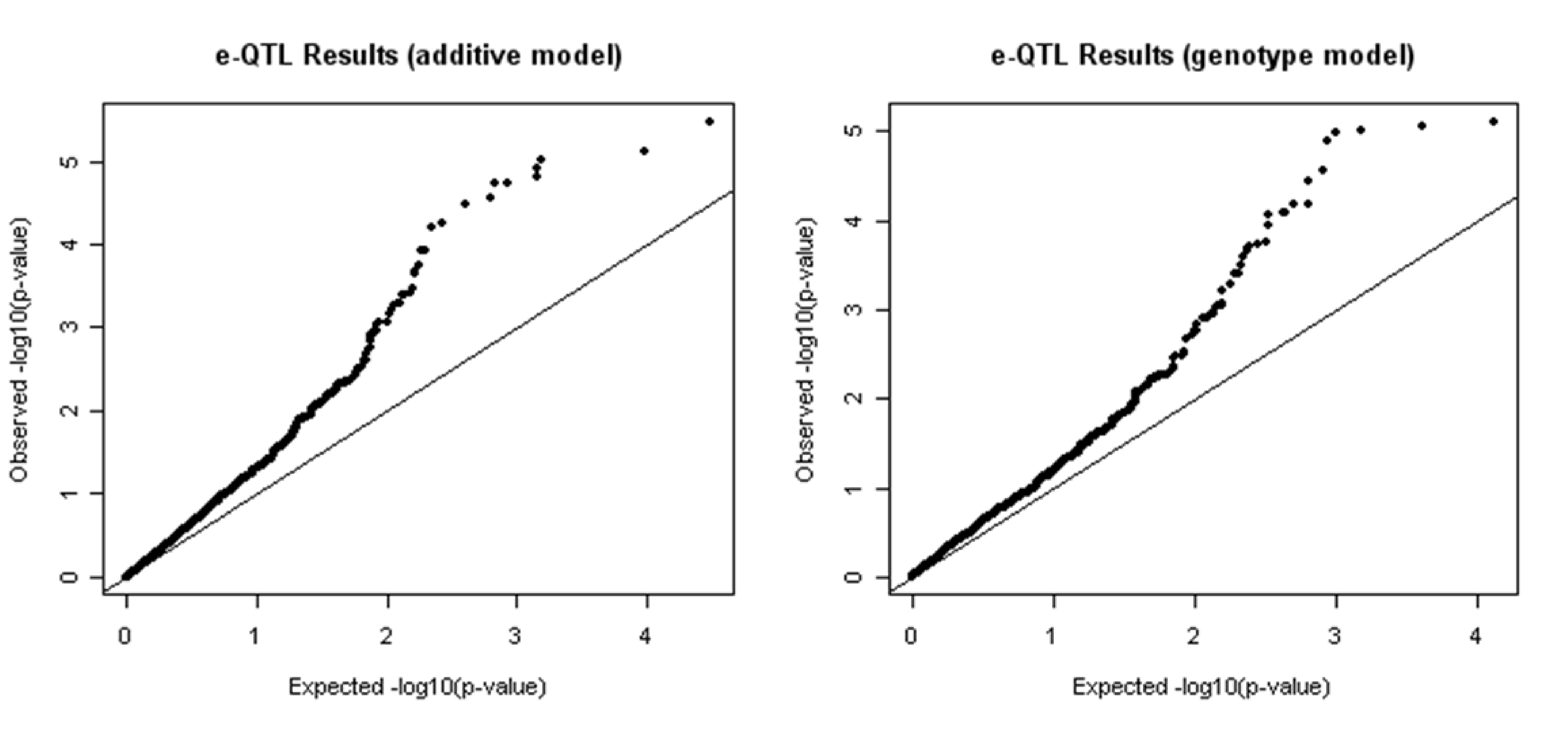

Supplement: Figure S1 — Illustrating the p-value distributions from different association tests. An eQTL analysis was performed using an additive (left) or genotype (middle) model. In both cases, there is enrichment of small p-values beyond what is expected due to chance. This enrichment is likely due to selection bias because both SNPs and genes were selected based on their association with self reported race. (0.87 MB TIF) [file pone.0008183.s001.tif]
